# HIV testing and linkage to ART following secondary distribution of HIV self‐test kits to male partners of women living with HIV: a pilot randomized control trial in Mpumalanga, South Africa

**DOI:** 10.1002/jia2.25937

**Published:** 2022-06-11

**Authors:** Dvora L. Joseph Davey, Kristin M. Wall, Nireshni Naidoo, Dhirisha Naidoo, Gugu Xaba, Claire Serao, Todd Malone, Kathryn Dovel

**Affiliations:** ^1^ Division of Infectious Diseases David Geffen School of Medicine University of California Los Angeles Los Angeles California USA; ^2^ Department of Epidemiology Fielding School of Public Health University of California Los Angeles Los Angeles California USA; ^3^ Division of Epidemiology and Biostatistics School of Public Health and Family Medicine University of Cape Town Cape Town South Africa; ^4^ Department of Epidemiology Rollins School of Public Health Emory University Atlanta Georgia USA; ^5^ BroadReach Healthcare Johannesburg South Africa

**Keywords:** HIV self‐testing, men, South Africa, index testing, women living with HIV, HIV testing

## Abstract

**Introduction:**

South African men are underrepresented in HIV testing and treatment services. Secondary distribution of oral HIV self‐test (HIVST) kits by women living with HIV (WLHIV) to their male partners (i.e. index partner HIVST) may increase men's testing and treatment but has been understudied.

**Methods:**

Between March and July 2021, we evaluated the effectiveness of index partner HIVST versus the standard of care (SOC) (invitations for men's facility‐based testing) on men's testing in a 1:1 randomized control trial. Eligibility criteria included: WLHIV; ≥18 years of age; attending one of four high‐density rural clinics; have a working cell phone; and self‐reported having a primary male partner of unknown serostatus. The primary outcome was the proportion of WLHIV reporting that her partner tested for HIV within 3 months after enrolment.

**Results:**

We enrolled 180 WLHIV and 176 completed an endline survey (mean age = 35 years, 15% pregnant, 47% unmarried or non‐cohabiting). In the HIVST arm, 78% of male partners were reported to have tested for HIV versus 55% in SOC (RR = 1.41; 95% CI = 1.14–1.76). In the HIVST arm, nine men were reactive with HIVST (14% positivity), six were confirmed HIV positive with standard testing (67%) and all of those started antiretroviral therapy (ART), and four HIV‐negative men started pre‐exposure prophylaxis (PrEP) (5%). In SOC, six men were diagnosed with HIV (12% positivity), 100% started ART and seven HIV‐negative men started PrEP (16%). One case of verbal intimate partner violence was reported in the HIVST arm.

**Conclusions:**

Secondary distribution of HIVST to partners of WLHIV was acceptable and effective for improving HIV testing among men in rural South Africa in our pilot study. Interventions are needed to link reactive HIVST users to confirmatory testing and ART.

## 1 INTRODUCTION

Though access to and uptake of HIV testing in South Africa has improved, men continue to lag behind cisgender women in South Africa [1]. In 2020, UNAIDS estimated that 87% of adult South African males living with HIV knew their serostatus (vs. 94% of females), 71% of those were on treatment (vs. 78% of females) and 65% of those were virally suppressed (vs. 72% of females), falling short of UNAIDS 90‐90‐90 targets, and far from reaching the 95‐95‐95 targets by 2030 [2]. The unmet need for HIV testing among men impedes efforts to bring the South African HIV epidemic under control [3, 4, 5]. Many men remain unaware of their HIV status until HIV‐related illness forces presentation at a clinic or health facility [6, 7, 8]. A primary reason for men's underrepresentation in HIV testing is limited access and opportunity to test—while women regularly engage with the health system and are frequently offered HIV testing through antenatal and children‐under five services [9], men must often actively seek out specific HIV clinics for testing [10]. Congested clinics, unfriendly staff, restricted clinic work hours, stigma and privacy concerns also act as barriers to men's use of clinic‐based HIV testing [11, 12]. Finally, there is qualitative evidence that harmful gender norms, such as believing men must be strong, self‐reliant and have multiple sex partners, may also deter men from testing [13]. Men residing in rural areas of South Africa face additional barriers to HIV services, including frequent migration to urban areas for work, and travelling far distances to health facilities in rural areas [14, 15].

A successful treatment cascade offers early and regular testing opportunities, linkage to HIV care for those who test HIV positive and client‐centred retention strategies to achieve undetectable viral loads [16, 17, 18]. Nkangala District, a predominantly peri‐urban and rural district in South Africa, has significant gaps in identifying people living with HIV (PLHIV) who are not already on antiretroviral therapy (ART), especially among adult men. In line with prior research, we estimate that over 65% of PLHIV not on ART in Nkangala District are men [1], which is a gap of ∼39,000 men living with HIV who need to be tested and initiated on treatment.

Index partner testing, defined as testing partners of individuals living with HIV, is a key strategy for reaching men [19, 20] but has not been brought to scale in Nkhangala District. Secondary distribution of HIV self‐testing kits (HIVST), whereby clients bring HIVST kits to their partners, can facilitate index partner testing by addressing many important barriers to testing [19, 21]. Individuals can use HIVST at their convenience and in the privacy of their own homes, removing most facility‐level barriers to care [22, 23]. Men perceive HIVST as time‐ and cost‐saving, and reported that this strategy reduces stigma and prepares them for retesting or confirmatory testing in the clinic [24]. However, secondary HIVST distribution has rarely been studied as part of index partner testing (i.e. index partner HIVST). Secondary HIVST distribution by female clients not living with HIV has increased male partner and couples testing in antenatal and postpartum care settings in Kenya [25], and male partner testing in antenatal care settings in Malawi [26]. Few adverse events, including intimate partner violence (IPV), have been associated with secondary HIVST distribution strategies in five randomized trials [27].

The one study that examined HIVST distribution among partners of individuals living with HIV found that male partner testing increased dramatically when using index partner HIVST with ART clients in Malawi [19]. However, the study population included women living with HIV (WLHIV) who were primarily in stable, long‐term partnerships where HIV status disclosure had largely already taken place [19]. Malawi's setting is in stark contrast to typical relationships in rural South Africa, where there is a high prevalence of unmarried/non‐cohabiting sexual relationships, migration and IPV [14, 15, 28]. It is critical to examine how HIVST can be incorporated as part of index partner testing in the rural South African context.

To improve case finding among men in rural South Africa, we conducted a randomized control trial to evaluate the feasibility and effectiveness of index partner HIVST by WLHIV compared to the standard of care (SOC) invitation for facility‐based HIV testing services (HTS).

## 2 METHODS

### 2.1 Study design and eligibility

Between March and July 2021, we conducted an individually randomized control trial among WLHIV across four health facilities. Participants had the following inclusion criteria: (1) female; (2) 18+ years old; (3) confirmed living with HIV (on or off of ART); (4) accessed health services at one of the four participating high‐burden health facilities; (5) reported having a primary male partner with whom they are sexually active and who is of HIV negative or unknown status; (6) had a cell phone that can read and respond to SMS/Whatsapp messages; and (7) were able to consent to study participation.

### 2.2 Study outcomes

WLHIV were followed for up to 3 months after study enrolment to ascertain the following study outcomes:

- Primary outcome: the proportion of WLHIV who report that her male partner tested for HIV during the study period
- Secondary outcomes: (1) positivity of men who tested, (2) linkage to ART among men with a reactive test or HIVST kit and (3) linkage to pre‐exposure prophylaxis (PrEP) among men who tested HIV negative.

### 2.3 Enrolment and randomization

**Figure 1:**
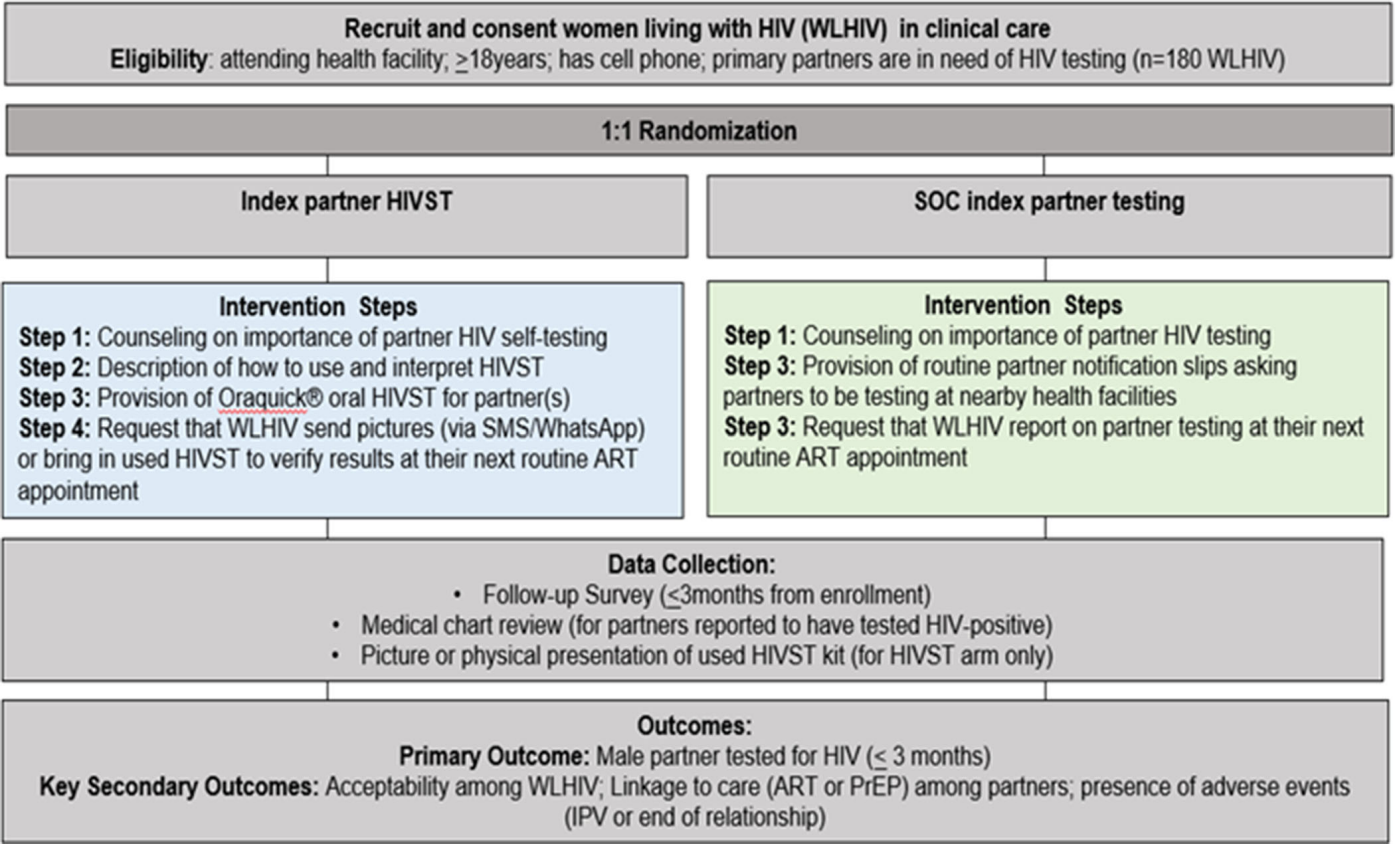
Study procedures of secondary distribution of HIV self‐tests (HIVST) by women living with HIV (WLHIV) in randomized control trial integrated into four public health clinics in Mpumalanga, South Africa.

**Figure 2:**
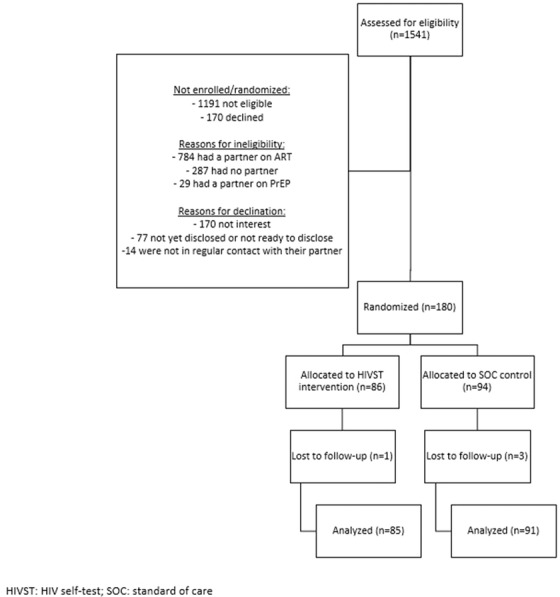
Flow of participants from eligibility assessment to data analysis in HIV self‐testing secondary distribution study among women living with HIV in Mpumalanga, South Africa.

The research team worked with clinic staff (nurses and HIV counsellors) to recruit and screen WLHIV who were attending HTS, HIV care/treatment services or antenatal care services in peri‐urban (*n* = 2) and rural (*n* = 2) high‐density facilities in Nkangala District. Trained study interviewers enrolled participants who were eligible and interested in the study. Participants were randomized 1:1 using a random number table by participant identifier (ID). All were randomized either to index partner HIVST or SOC referral for facility‐based HIV testing and counselling (Figure 1). Those who were eligible but declined study participation were considered as refused study participation (see consort flow diagram, Figure 2).

### 2.4 Intervention

Following randomization, WLHIV in the intervention arm received counselling on the use of oral HIVST and how best to explain the HIVST kit, and importance of HIV testing, for her partner(s). The study counsellor explained to women how to interpret the results, using the package insert of the OraQuick test, and the importance of verification of test result if reactive (two lines) or if indeterminate (no lines). Participants were encouraged to call the study phone number to get support in the case of problems with the test interpretation or difficulty with their male partners reaction to the test. Female participants were encouraged to send a picture of the used HIVST to the study counsellor, or to bring the used HIVST in their next clinical follow‐up visit and study visit, which was scheduled at the same date.

### 2.5 Standard of care

WLHIV in the SOC arm received counselling on the importance of their partner testing for HIV and were given a physical referral slip to give to her partner to encourage her partner(s) to get tested at their nearby facility. Women were asked to report partner testing to the study team in their next clinical follow‐up visit.

### 2.6 Data collection

After consent and implementing the intervention (either index partner HIVST or SOC), the same trained study interviewer administered a brief 10–15 minute baseline survey about socio‐demographics (age, education and relationship status), knowledge of partner's prior HIV testing and status, sexual behaviours and IPV [29]. All surveys were conducted in the local language (isiZulu or Tsonga) using RedCap.

During participants’ next regular clinical visit (up to 3 months since enrolment), trained study interviewers conducted an endline survey that documented WLHIV self‐reports on primary and secondary study outcomes. If the participant did not return to the clinic, they were interviewed by telephone by the interviewer. Endline measures included partner's use of HTS, acceptability of the intervention and any adverse events within the study period. Acceptability measures included ability of participants to deliver the intervention (HIVST or partner referral slips), and describe the HIVST kit to their partner (in the HIVST arm only). In addition, the survey included questions on barriers to the intervention and any reported harms or reactions during the study period. In the intervention arm, participants were requested to validate the use of the HIVST by taking and sending a photo of the used kit via SMS or WhatsApp or returning the used test kit(s) in their study visit. All participants enrolled were provided with R50 (∼$3USD) in airtime credit for each study visit ($6USD total).

Study staff conducted medical chart reviews to record the use of facility‐based HIV testing (in both arms), and PrEP and ART initiation at the end of the study. They also collected viral suppression records for participating WLHIV who were actively taking ART and any male partner who initiated ART.

### 2.7 Data analysis

We followed CONSORT standards [30] for all analyses and reporting. Data analyses were conducted using SAS version 9.4 [31]. Among women assessed for study eligibility, we describe the number of who were ineligible, who refused to participate and who were enrolled and randomized. Among those randomized, we describe loss to follow‐up along with participants’ median (and interquartile range [IQR]) follow‐up time. Baseline demographic characteristics are described overall and stratified by study arm to explore possible failures of randomization using frequencies for categorical variables and means/standard deviations (SD) or median/IQRs as appropriate depending on variable distribution.

In our primary analysis, we conducted an intention‐to‐treat analysis and tabulated the proportion of primary and secondary outcomes by study arm. We used a binomial model to output risk ratios and 95% confidence intervals. We also conducted two secondary analyses of the primary outcome: a per‐protocol analysis and an analysis adjusting for baseline factors that were imbalanced by study arm. Finally, we quantitatively describe experiences and acceptability of women who distributed the HIVST as well as experiences and acceptability of men who used the HIVST.

### 2.8 Power calculations

We aimed to enrol 100 participants per arm based on feasibility and funding available for the pilot study. Based on post hoc analysis, with our final sample size, we were powered to detect the observed primary outcome effects with >90% power.

### 2.9 Ethics

Written informed consent was attained by all participants. Participant consent included consent for abstraction of participant data from the facilities. The study was reviewed and approved by the University of Cape Town Human Research Ethics Committee (#697‐2020) and conforms to standards currently applied in South Africa. University of California Los Angeles relied on the University of Cape Town Ethics review.

## 3 RESULTS

### 3.1 Participant flow

Of the 1541 women who were assessed for eligibility, *n* = 1100 (71%) were not eligible (Figure 1). Of the 1541 women who were assessed for eligibility, *n* = 261 (17%) refused to enrol. The remaining *n* = 180 were randomized to the index partner HIVST arm (*n* = 86) or the SOC control arm (*n* = 94). The majority of participants were retained in the study until endline (99% in the HIVST arm and 97% in the SOC arm), and our final analysis sample included *n* = 85 women in the HIVST arm and *n* = 91 women in the SOC arm. Mean follow‐up time for participants was 32 days (SD = 22 days), with a median of 32 (IQR = 32) days and range of 0–92 days. Follow‐up time did not differ by study arm.

### 3.2 Baseline demographics and randomization

The average age of study participants was 35 years (SD = 9 years) and most WLHIV (56%) had completed some tertiary schooling (Table 1). Over half of participants (52%) were unemployed and 41% reported no monthly household income. At enrolment, most women were using ART (94%), were virally supressed (88%) and reported having disclosed to their primary partner (88%) and family members (70%), and there were no differences in these characteristics by study arm.

**Table 1:** Baseline demographics, sexual behaviours, and HIV testing acceptability and barriers, by study arm in randomized control trial of secondary HIV testing by women living with HIV in South Africa (*n* = 176)

		Total (*N* = 176)		HIVST (*N* = 85)		SOC (*N* = 91)	
		*N*	%	*N*	%	*N*	%
Demographics							
Age (years), mean SD		34.8	855	33.8	8.6	35.7	8.5
Highest level of education completed	None	6	3%	5	6%	1	1%
	Completed primary school	4	2%	1	1%	3	3%
	Some secondary school	19	11%	8	9%	11	12%
	Completed secondary school	48	27%	23	27%	25	27%
	Some tertiary school	98	56%	48	56%	50	55%
	Degree/diploma from tertiary school	1	1%	0	0%	1	1%
Current employment status	Employed full‐time	62	35%	28	33%	34	37%
	Employed part‐time	14	8%	9	11%	5	5%
	Self‐employed	8	5%	2	2%	6	7%
	Not employed	92	52%	46	54%	46	51%
Monthly household income	None	72	41%	35	41%	37	41%
	<$150/month	31	18%	17	20%	14	15%
	$150–300/month	31	18%	14	16%	17	19%
	$300–500/month	32	18%	13	15%	19	21%
	>$500/month	10	6%	6	7%	4	4%
Number of biological children, mean SD		1.8	1.2	1.7	1.2	1.9	1.3
Currently pregnant		26	15%	12	14%	14	16%
Relationship status with primary partner	Married	41	23%	12	14%	29	32%
	Steady partner living with me	52	30%	29	34%	23	25%
	Steady partner not living with me	66	38%	33	39%	33	36%
	Casual partner	17	10%	11	13%	6	7%
Length of relationship with partner (months), median IQR		48	50	48	48	48	72
Current employment status of primary partner	Employed full‐time	121	69%	60	71%	61	67%
	Employed part‐time	22	13%	9	11%	13	14%
	Self‐employed	20	11%	8	10%	12	13%
	Not employed	12	7%	7	8%	5	5%
Currently on ART		167	94%	81	95%	86	93%
Virally suppressed (VL <1000 copies)		140	84%	68	84%	72	84%
Disclosed HIV status to whom? (Select all that apply)	Partner	154	88%	74	87%	80	88%
	Friends	35	20%	14	16%	21	23%
	Family member	123	70%	63	74%	60	66%
	No one (not disclosed)	6	3%	4	5%	2	2%
Sexual behaviours							
Condom used at last sex		78	44%	40	47%	38	42%
>1 sex partner in the past year		27	15%	14	16%	13	14%
In the past year, has your PRIMARY SEX partner:							
insulted or yelled at you?		3	2%	1	1%	2	2%
threatened to hurt you or someone you care about?		2	1%	1	1%	1	1%
pushed, shoved, kicked, hit or beaten you up?		3	2%	2	2%	1	1%
threated to use a gun, knife or weapon against you?		1	1%	1	1%	0	0%
forced you to have sex with him?		1	1%	1	1%	0	0%

Abbreviations: ART, antiretroviral treatment; HIVST, HIV self‐test; SOC, standard of care; SD, standard deviation.

Women had on average 1.8 children (SD = 1.2) and 15% were pregnant at enrolment. Many women reported not cohabiting with their partner (38%) or having a casual sex partner (10%), and 30% were cohabiting but unmarried. More women in the SOC arm reported being married than in the HIVST arm (34% vs. 14%), while more women in the HIVST arm reported living with a steady partner or having a casual partner relative to the SOC arm (34% vs. 25% and 13% vs. 7%, respectively). Women reported being in a relationship with their primary partner for a median of 4 years (IQR = 4 years), and the majority of primary partners were employed full‐time (69%). Over half of women (56%) reported not using a condom during last sex, and 15% reported having outsider partners in the past year. Very few (<2%) participants reported that they had experienced IPV in the past year with their primary partner.

### 3.3 Outcomes

**Figure 3:**
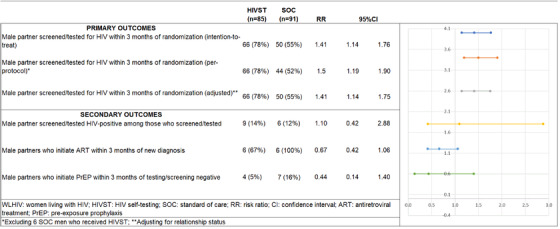
Outcomes of randomized control trial of secondary distribution of HIV self‐testing by women living with HIV to male partners in Mpumalanaga, South Africa (*n* = 176).

A higher proportion of male partners tested for HIV within 3 months after study enrolment in the HIVST versus SOC arm (78% vs. 55%, RR = 1.41, 95% CI: 1.14, 1.76, *p* = 0.002) in the intention‐to‐treat analysis (Figure 3). This statistically significant association persisted in per‐protocol analyses, which excluded six men from the SOC arm who inadvertently received an HIVST (RR = 1.50, 95% CI: 1.19, 1.90, *p* = 0.01) and after adjusting for relationship status (adjusted RR = 1.41, 95% CI: 1.14–1.75, *p* = 0.01). A similar number of men tested positive for HIV by study arm (14% HIVST vs. 12% SOC, RR = 1.10, 95% CI: 0.42, 2.88, *p* = 0.85). However, in the HIVST arm, fewer male partners initiated ART within 3 months of study enrolment as compared to SOC (67% vs. 100%, RR = 0.67, 95% CI: 0.42–1.06, *p* = 0.37) and fewer male partners initiated PrEP within 3 months of study enrolment in the HIVST versus SOC arm (5% vs. 16%, RR = 0.44, 95% CI: 0.14–1.40, *p* = 0.267).

### 3.4 HIVST distribution

Sixty‐eight of the 85 women in the HIVST arm (80%) reported having distributed the HIVST kit to their sexual partner (Table 2). Among those 68 women, most (54%) distributed HIVST kits within a week after enrolling in the study. Women's comfort with HIVST kit distribution was high (96% were comfortable or very comfortable), and 94% of WLHIV reported demonstrating how to use the kit to their partners. Very few women (6%) reported pressuring their partner to use the HIVST.

**Table 2:** Self‐reported data from index partners in the intervention arm who gave the HIVST to their partner in randomized control trial of secondary HIV testing by women living with HIV in South Africa (*n* = 68 in intervention arm)

		WLHIV who distributed the HIVST (*N* = 68)	
		*N*	%
When did you give your partner the HIV self‐test?	Same day as receipt	12	18%
	1–7 days later	10	15%
	8–14 days later	15	22%
	15+ days later	22	32%
	Don't remember	9	13%
How comfortable were you describing the HIV self‐test kit?	Very comfortable	12	18%
	Comfortable	53	78%
	Uncomfortable	3	4%
Did you demonstrate how to use the HIV self‐test?	Yes	64	94%
	No	4	6%
Did you pressure him to use the HIVST?	Yes	4	6%
	No	64	94%
How do you know your partner used the HIVST, among those whose partners tested? (Select all that apply)	Index self‐reported that her partner told her	6	9%
	Picture of the used HIVST sent by index via SMS/WhatsApp	1	2%
	Picture of the used HIVST presented at facility	10	15%
	Used HIVST brought to facility by the index	35	53%
	Index partner self‐reports being with male partner when he used the HIVST	12	18%
How satisfied was your partner with receiving the HIV self‐test?	Very satisfied	10	15%
	Satisfied	54	79%
	Dissatisfied	1	1%
	Very dissatisfied	2	3%
How difficult was it for your partner to use the test in your opinion?	Not difficult at all	58	87%
	A little difficult	5	7%
	Difficult	1	1%
	Very difficult	0	0%
	Don't know	3	4%
Did your partner find out their result?	Yes	65	97%
	No	1	1%
	Don't know	1	1%
Do you know your partner's result?	Yes, negative	56	84%
	Yes, positive	9	13%
	Don't know	2	3%
Did you disclosure your HIV result to your partner?	Yes, he already knew (before this study)	51	76%
	Yes, told him when I gave him the HIVST	13	19%
	Yes, we used HIV self‐tests together and shared our results	1	1%
	Yes, we both got tested together in the clinic and shared our results	0	0%
	No	2	3%
If yes, how was that experience of disclosing to your partner?	Very good	22	33%
	Good	43	65%
	Bad/very bad/uncomfortable	1	2%
Has your partner insulted you or yelled at you since you gave him the HIVST?	Yes	1	1%
	No	67	99%

Abbreviations: HIVST, HIV self‐test; WLHIV, women living with HIV.

HIVST use was primarily verified by women bringing the used HIVST to the endline survey (53%) or sending a picture of the used test to study staff prior to the endline survey (15%). Only a minority of WLHIV were unable to show the used HIVST kit to study staff. Women believed that their male partners were highly satisfied with the HIVST intervention (96%) and very few believed their partner had difficulty using the kit (13%). Most women reported that their male partner was able to interpret the HIVST result (97%) and the same proportion reported that they knew their partners HIVST result (85% negative and 13% positive).

Over three‐fourths (76%) of WLHIV reported that they had previously disclosed their HIV status to their male partner prior to enrolling in the study. Of those who had not already disclosed, 19% reported that they disclosed when distributing the HIVST kit to their partner or they used the HIVST together or shared their result (1%), and two women reported that they did not disclose (3%).

One woman reported that her partner insulted and yelled at her and that this event was due to distributing the HIVST. No women reported that their partner threatened to hurt or harm them or someone they cared about or pushed, shoved, kicked, hit or beat her up. No women reported giving the HIVST to someone other than their partner.

## 4 DISCUSSION

Innovative HIV testing strategies are needed for men in rural South Africa, where men account for the majority of individuals living with HIV but not on treatment [1]. We conducted one of the first studies to examine the feasibility and effectiveness of secondary distribution of HIVST to partners of WLHIV in rural South Africa (i.e. index partner testing). Our study is unique because our population in Nkangala District consisted largely of WLHIV who were unmarried or not cohabiting with their male partner. Our study demonstrates that using secondary distribution of HIVST to facilitate index partner testing increased men's uptake of testing services, with 78% male partners tested in the HIVST arm compared to 55% in the standard HTS arm. The majority of women verified this HIVST kit use with pictures or bringing the actual kit. Importantly, no physical adverse events were reported by WLHIV, suggesting that HIVST for index partner testing may be a feasible, safe strategy to reach men, even in mobile settings and within non‐cohabiting relationships. These findings are in line with another study from Malawi that shows index partner HIVST is feasible and acceptable to WLHIV [22].

The majority of women believed that index partner HIVST was acceptable to their male partner, similar to other literature that unanimously report HIVST to be acceptable and desired by men [22, 32, 33, 34]. Our findings suggest that index partner HIVST may be implemented with fidelity and with little to no harm to WLHIV, even among communities with non‐cohabiting and possibly more mobile populations. Secondary HIVST distribution across the region continues to show very low risk of harm [27, 35], even among sex workers in Kenya and Zambia, despite the insecure nature and often unequal power dynamics of their sexual relationships [25, 36]. The feasibility of secondary HIVST across relationship types and varying relationship power dynamics may indicate that women are able to self‐identify when distributing HIVST may increase the risk of harm and either not distribute kits to this partner, or have a strategy to navigate HIVST distribution that minimizes risk. A recent study in South Africa finds that only a small proportion of adolescent girls and young women (18–26 years) distributed HIVST to their sexual partners—the vast majority of partnerships were unmarried [37], suggesting that a combination of age and relationship dynamics may influence perceived risk and benefit of HIVST distribution. Importantly, very few WLHIV in our study reported IPV at baseline, meaning that women at risk of IPV may have opted‐out of the study. Larger trials are needed to fully understand the risk of adverse events (such as IPV and end of relationship) among this population, and characteristics of WLHIV who opt‐out of index partner HIVST.

A small number of participants who had not previously disclosed their HIV status to their partner used HIVST as a tool for disclosure. Status disclosure may promote treatment adherence and increased support among WLHIV, although this has not been tested within the context of index partner HIVST. The potential of secondary benefits from index partner HIVST deserves further investigation. Importantly, over 50% of WLHIV who distributed HIVST to their partners did so more than a week after receiving the HIVST kit. Delays in HIVST distribution may be due to men's absence due to work and/or travel, or simply not seeing partners daily as many WLHIV reported non‐cohabitating relationships, although this deserves further exploration to confirm our hypothesis.

Among men who tested for HIV, positivity rates were similar across arms, with 14% (9/66) positivity in the HIVST arm compared to 12% (6/50) in the standard HTS arm. ART initiation among those diagnosed as HIV positive was 67% (6/9) and 100% (6/6) within HIVST and standard HTS arms, respectively. While the sample size is small, findings corroborate with other literature that shows lower rates of ART initiation among those using secondary HIVST distribution models [22]. ART initiation in men who were screened at home may be lower due to fears of stigma, and logistical barriers of coming into the clinic especially during the week while working or in a migratory population. This is not surprising since the use of HIVST does not remove barriers to ART initiation—the same barriers overcome by HIVST still must be faced for men now needing to link to care and start treatment. Barriers may be particularly salient from our study population given the high levels of migration among men in rural South Africa and, therefore, may not be able to access their local health facility even if they wanted to initiate ART [15, 38]. Future studies should examine how to combine secondary HIVST with linkage to care strategies to improve ART initiation among men who test positive.

This pilot trial has several strengths and limitations. First, our study had excellent retention considering that this was integrated into busy primary healthcare facilities. This is due to having trained research staff and flexible study visits (done by telephone or during other clinical visits or drug pickups). Second, the majority of women verified HIVST kit use with pictures or bringing the actual kit, increasing the likelihood that secondary reports were accurate. In terms of limitations, baseline and follow‐up surveys were conducted by study and facility staff who were also implementing the HIVST and standard HTS interventions and may be sensitive to social desirability bias. As a result, participants may have felt increased pressure to report positive HIVST outcomes, and some of the study staff gave some control participants an HIVST kit based on perceived need. We removed these cases from our intention‐to‐treat analysis and found no meaningful differences in results. Further, we relied on a small sample size and may not be able to detect potential risk of infrequent adverse events, such as IPV. The screen to enrol ratio in this study was high (8.6:1), mostly due to ineligibility. The main reasons for study ineligibility were having a partner already on ART or reporting having no partner. The time and effort spent on screening should be a consideration for future programs that largely recruit women from ART services. Finally, our study was not powered to detect differences in positivity and linkage to ART. Additional research is needed to assess if similar findings can be replicated in other health facilities and other districts where non‐cohabiting relationships and mobile populations are common.

## 5 CONCLUSIONS

Index partner HIVST shows promise to increase men's use of HIV testing among South African populations at higher risk of HIV infection (i.e. partners of WLHIV) and with a high prevalence of non‐cohabitating relationships. Additional research is needed on the potential of secondary benefits to index partner HIVST, such as increased disclosure and better treatment adherence among WLHIV, understanding the characteristics of WLHIV who opt‐out of index partner HIVST and how to improve the linkage to ART among men using HIVST.

## COMPETING INTERESTS

There are no competing interests.

## AUTHORS’ CONTRIBUTIONS

DLJD designed the study, managed the study implementation, cleaned and analysed the data and wrote the first draft of the manuscript. KMW designed the study, analysed the data, conducted all analysis and revised the drafts of the manuscript. NN, DN, GX and CS oversaw study and programmatic implementation, data collection, data cleaning and reviewed/approved of the manuscript. TM provided oversight for the study and reviewed/approved of the manuscript. KD designed the study, wrote the first drafts of the manuscript and revised drafts of the manuscript.

## Data Availability

Deidentified data will be made available by contacting the study PI, djosephdavey@mednet.ucla.edu.

## References

[1] Human Science Research Council (HSRC) . South African National Prevalence, Incidence, Behaviour and Communication Survey. 2018. http://wwwhsrcacza/en/media‐briefs/hiv‐aids‐stis‐and‐tb/sabssm‐launch‐2018v2. Accessed November 1, 2022

[2] UNAIDS . UNAIDS Data 2020. Geneva; 2021.

[3] Hoffman CM , Fritz L , Matlakala N , Mbambazela N , Railton JP , McIntyre JA , et al. Community‐based strategies to identify the unmet need for care of individuals with sexually transmitted infection‐associated symptoms in rural South Africa. Trop Med Inter Health. 2019;24(8):987–93. 10.1111/tmi.13274 31141301

[4] Smith PJ , Davey DJ , Green H , Cornell M , Bekker L‐G . Reaching underserved South Africans with integrated chronic disease screening and mobile HIV counselling and testing: a retrospective, longitudinal study conducted in Cape Town. PLoS One. 2021;16(5):e0249600. 33945540 10.1371/journal.pone.0249600 PMC8096085

[5] Wade AN , Payne CF , Berkman L , Chang A , Gómez‐Olivé FX , Kabudula C , et al. Multimorbidity and mortality in an older, rural black South African population cohort with high prevalence of HIV findings from the HAALSI Study. BMJ Open. 2021;11(9):e047777. 10.1136/bmjopen-2020-047777 PMC8444254 34526338

[6] Lilian RR , Rees K , Mcintyre JA , Struthers HE , Peters RPH . Same‐day antiretroviral therapy initiation for HIV‐infected adults in South Africa: analysis of routine data. PLoS One. 2020;15(1):e0227572. 31935240 10.1371/journal.pone.0227572 PMC6959580

[7] Hirasen K , Fox MP , Hendrickson CJ , Sineke T , Onoya D . HIV treatment outcomes among patients initiated on antiretroviral therapy pre and post‐universal test and treat guidelines in South Africa. Ther Clin Risk Manag. 2020;16:169–80. 32184609 10.2147/TCRM.S227290 PMC7061415

[8] Ware NC , Wyatt MA , Pisarski EE , Bwana BM , Orrell C , Asiimwe S , et al. Influences on adherence to antiretroviral therapy (ART) in early‐stage HIV disease: qualitative study from Uganda and South Africa. AIDS Behav. 2020;24(9):2624–2636. 10.1007/s10461-020-02819-z. PMID: 32140877 32140877 PMC11091710

[9] Yeatman S , Chamberlin S , Dovel K . Women's (health) work: a population‐based, cross‐sectional study of gender differences in time spent seeking health care in Malawi. PLoS One. 2018;13(12):e0209586. 30576388 10.1371/journal.pone.0209586 PMC6303093

[10] Dovel K , Dworkin SL , Cornell M , Coates TJ , Yeatman S . Gendered health institutions: examining the organization of health services and men's use of HIV testing in Malawi. J Inter AIDS Soc. 2020;23:e25517. 10.1002/jia2.25517 PMC7319160 32589346

[11] Bassett IV , Coleman SM , Giddy J , Bogart LM , Chaisson CE , Ross D , et al. Barriers to care and 1‐year mortality among newly diagnosed HIV‐infected people in Durban, South Africa. J Acquir Immune Defic Syndr. 2017;74(4):432–8. 28060226 10.1097/QAI.0000000000001277 PMC5321110

[12] Clouse K , Pettifor AE , Maskew M , Bassett J , Van Rie A , Behets F , et al. Patient retention from HIV diagnosis through one year on antiretroviral therapy at a primary health care clinic in Johannesburg, South Africa. J Acquir Immune Defic Syndr. 2013;62(2):e39–46. 23011400 10.1097/QAI.0b013e318273ac48 PMC3548953

[13] Sileo KM , Fielding‐Miller R , Dworkin SL , Fleming PJ . What role do masculine norms play in men's HIV testing in sub‐Saharan Africa?: a scoping review. AIDS Behav. 2018;22(8):2468–79. 29777420 10.1007/s10461-018-2160-z PMC6459015

[14] Dzomba A , Tomita A , Govender K , Tanser F . Effects of migration on risky sexual behavior and HIV acquisition in South Africa: a systematic review and meta‐analysis, 2000–2017. AIDS Behav. 2019;23(6):1396–30. 30547333 10.1007/s10461-018-2367-z

[15] Lurie MN , Williams BG , Zuma K , Mkaya‐Mwamburi D , Garnett GP , Sturm AW , et al. The impact of migration on HIV‐1 transmission in South Africa: a study of migrant and nonmigrant men and their partners. Sex Transm Dis. 2003;30(2):149–56. 12567174 10.1097/00007435-200302000-00011

[16] Pascoe SJ , Fox MP , Huber AN , Murphy J , Phokojoe M , Gorgens M , et al. Differentiated HIV care in South Africa: the effect of fast‐track treatment initiation counselling on ART initiation and viral suppression as partial results of an impact evaluation on the impact of a package of services to improve HIV treatment adherence. J Int AIDS Soc. 2019;22(11):e25409. 31691521 10.1002/jia2.25409 PMC6831947

[17] Ahmed S , Autrey J , Katz IT , Fox MP , Rosen S , Onoya D , et al. Why do people living with HIV not initiate treatment? A systematic review of qualitative evidence from low‐ and middle‐income countries. Soc Sci Med. 2018;213:72–84. 30059900 10.1016/j.socscimed.2018.05.048 PMC6813776

[18] Enane LA , Davies M‐A , Leroy V , Edmonds A , Apondi E , Adedimeji A , et al. Traversing the cascade: urgent research priorities for implementing the ‘treat all’ strategy for children and adolescents living with HIV in sub‐Saharan Africa. J Virus Erad. 2018;4(2):40–6. 30515313 10.1016/S2055-6640(20)30344-7 PMC6248846

[19] Dovel K , Balakasi K , Shaba F , Phiri K , Offorjebe O , Gupta S , et al. A randomized trial on index HIV self‐testing for sexual partners of ART clients in Malawi. Conference on Retroviruses and Opportunistic Infections (CROI); March 4–7, 2019; Seattle, Washington; March 4–7, 2019.

[20] Joseph Davey D , Wall KM , Serrao C , Prins M , Feinberg M , Mtonjana N , et al. HIV positivity and referral to treatment following testing of partners and children of PLHIV index patients in public sector facilities in South Africa. J Acquir Immune Defic Syndr. 2019;81(4):365–70. 30973546 10.1097/QAI.0000000000002048 PMC6637406

[21] Offorjebe OA , Hoffman RM , Shaba F , Balakasi K , Davey DJ , Nyirenda M , et al. Acceptability of index partner HIV self‐testing among HIV‐positive clients in Malawi: a mixed methods analysis. PLoS One. 2020;15(7):e0235008. 32649664 10.1371/journal.pone.0235008 PMC7351183

[22] Jamil MS , Eshun‐Wilson I , Witzel TC , Siegfried N , Figueroa C , Chitembo L , et al. Examining the effects of HIV self‐testing compared to standard HIV testing services in the general population: a systematic review and meta‐analysis. E Clin Med. 2021;38:100991. 10.1016/j.eclinm.2021.100991 PMC8271120 34278282

[23] Eshun‐Wilson I , Jamil MS , Witzel TC , Glidded DV , Johnson C , Trouneau NL , et al. A systematic review and network meta‐analyses to assess the effectiveness of human immunodeficiency virus (HIV) self‐testing distribution strategies. Clin Infect Dis. 2021;73(4):e1018–28. 34398952 10.1093/cid/ciab029 PMC8366833

[24] Stevens DR , Vrana CJ , Dlin RE , Korte JE . A global review of HIV self‐testing: themes and implications. AIDS Behav. 2018;22(2):497–512. 28155039 10.1007/s10461-017-1707-8 PMC5910655

[25] Masters SH , Agot K , Obonyo B , Napierala Mavedzenge S , Maman S , Thirumurthy H . Promoting partner testing and couples testing through secondary distribution of HIV self‐tests: a randomized clinical trial. PLoS Med. 2016;13(11):e1002166. 27824882 10.1371/journal.pmed.1002166 PMC5100966

[26] Choko AT , Corbett EL , Stallard N , Maheswaran H , Lepine A , Johnson CC , et al. HIV self‐testing alone or with additional interventions, including financial incentives, and linkage to care or prevention among male partners of antenatal care clinic attendees in Malawi: an adaptive multi‐arm, multi‐stage cluster randomised trial. PLoS Med. 2019;16(1):e1002719. 30601823 10.1371/journal.pmed.1002719 PMC6314606

[27] Johnson CC , Kennedy C , Fonner V , Siegfried N , Figueroa C , Dalal S , et al. Examining the effects of HIV self‐testing compared to standard HIV testing services: a systematic review and meta‐analysis. J Int AIDS Soc. 2017;20(1):21594. 28530049 10.7448/IAS.20.1.21594 PMC5515051

[28] Andrews JR , Wood R , Bekker L‐G , Middelkoop K , Walensky RP . Projecting the benefits of antiretroviral therapy for HIV prevention: the impact of population mobility and linkage to care. J Infect Dis. 2012;206(4):543–51. 22711905 10.1093/infdis/jis401 PMC3491737

[29] Harichund C , Moshabela M . Acceptability of HIV self‐testing in sub‐Saharan Africa: scoping study. AIDS Behav. 2018;22(2):560–8. 28699017 10.1007/s10461-017-1848-9 PMC5764831

[30] CONSORT 2010 Statement: updated guidelines for reporting parallel group randomized trials. Ann Inter Med. 2010;152(11):726–32. 10.7326/0003-4819-152-11-201006010-00232 20335313

[31] S. I. Inc . SAS/ACCESS® 9.4 Interface 2013.

[32] Indravudh PP , Fielding K , Chilongosi R , Nzawa R , Neuman M , Kumwenda MK , et al. Effect of door‐to‐door distribution of HIV self‐testing kits on HIV testing and antiretroviral therapy initiation: a cluster randomised trial in Malawi. BMJ Glob Health. 2021;6(4):e004269. 10.1136/bmjgh-2020-004269. PMID: 34275866; PMCID: PMC8287599. PMC8287599 34275866

[33] Sibanda EL , Neuman M , Tumushime M , Mangenah C , Hatzold K , Watadzaushe C , et al. Community‐based HIV self‐testing: a cluster‐randomised trial of supply‐side financial incentives and time‐trend analysis of linkage to antiretroviral therapy in Zimbabwe. BMJ Glob Health. 2021;6(4). 10.1136/bmjgh-2020-003866 PMC8287602 34275865

[34] Majam M , Conserve DF , Zishiri V , Haile ZT , Tembo A , Phiri J , et al. Implementation of different HIV self‐testing models with implications for HIV testing services during the COVID‐19 pandemic: study protocol for secondary data analysis of the STAR Initiative in South Africa. BMJ Open. 2021;11(5):e048585. 10.1136/bmjopen-2020-048585 PMC8130734 34006558

[35] Kumwenda MK , Johnson CC , Choko AT , Lora W , Sibande W , Sakala D , et al. Exploring social harms during distribution of HIV self‐testing kits using mixed‐methods approaches in Malawi. J Int AIDS Soc. 2019;22(1):e25251. 30907508 10.1002/jia2.25251 PMC6432111

[36] Pintye J , Drake AL , Begnel E , Kinuthia J , Abuna F , Lagat H , et al. Acceptability and outcomes of distributing HIV self‐tests for male partner testing in Kenyan maternal and child health and family planning clinics. AIDS. 2019;33(8):1369–78. 30932954 10.1097/QAD.0000000000002211 PMC6546533

[37] Pettifor A , Lippman SA , Kimaru L , Haber N , Mayakayaka Z , Selin A , et al. HIV self‐testing among young women in rural South Africa: a randomized controlled trial comparing clinic‐based HIV testing to the choice of either clinic testing or HIV self‐testing with secondary distribution to peers and partners. EClinicalMedicine. 2020;21:100327. 32322811 10.1016/j.eclinm.2020.100327 PMC7171186

[38] Ambia J , Kabudula C , Risher K , Gómez‐Olivé FX , Rice BD , Etoori D , et al. Outcomes of patients lost to follow‐up after antiretroviral therapy initiation in rural north‐eastern South Africa. Trop Med Int Health. 2019;24(6):747–56. 30920699 10.1111/tmi.13236 PMC6563456

